# Improvement of phytochemical and quality characteristics of *Dracocephalum kotschyi* by drying methods

**DOI:** 10.1002/fsn3.3351

**Published:** 2023-04-26

**Authors:** Sahar Zamani, Davood Bakhshi, Amir Sahraroo, Mohammad‐Taghi Ebadi

**Affiliations:** ^1^ Department of Horticultural Science, Faculty of Agricultural Sciences University of Guilan Rasht Iran; ^2^ Department of Horticultural Science, Faculty of Agriculture Tarbiat Modares University Tehran Iran

**Keywords:** antioxidant, essential oil, infrared drying, microbial contamination, refractance window drying

## Abstract

This experiment was conducted to evaluate the effects of different drying methods on drying parameters and qualitative characteristics of *Dracocephalum kotschyi* in a completely randomized design with three replications. Treatments included shade drying as control, sun drying, cabinet drying (CD at 50 and 60°C), refractance window drying (RWD), infrared drying (IRD) at 200 and 300 W, and combination of RWD+ IRD at 200 and 300 W. According to the results, IRD, RWD, and RWD+ IRD effectively maintained valuable secondary metabolites compared to the conventional drying methods. The maximum total phenol content (2.7 and 2.66 mg GAE/g dry weight), total flavonoid content (2.26 and 2.33 mg QE/g dry weight), antioxidant activity (79% and 78.33%), and essential oil content (0.65% and 0.76%) were obtained from plants dried by RWD and IRD. Samples dried by RWD, IRD, and RWD+ IRD had high color quality, acceptable green color, and less browning. Also, RWD and IRD methods effectively reduced microbial contamination of dried plants compared to the control and other methods. The minimum aerobic mesophiles, mold, yeast, and coliforms were observed at 3.11, 0, and 1.47 log CFU/g in IRD 300 W and 3.17, 1, and 1.30 log CFU/g in RWD. *D. kotschyi* dried at CD 50°C had the maximum microbial contamination. Generally, according to the obtained results, RWD and IRD methods are suggested for drying of D. kotschyi and similar herbs due to shortening the drying time, preserving and improving the quality properties of dried plants.

## Graphical abstract

The research was conducted for the first time to compare various drying methods (refractance window drying (RWD), infrared drying (IRD), and RWD+ IRD in comparison to conventional drying methods) to select the appropriate method for *Dracocephalum kotschyi* with an emphasis on protecting the phytochemical characteristics accompanied by reducing microbial contamination.

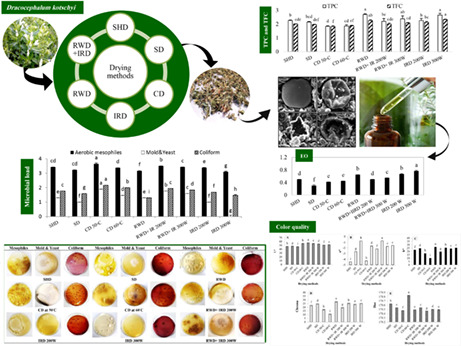

## 1 INTRODUCTION

Medicinal and aromatic plants (MAPs) are used in pharmaceutical, food, cosmetic, and other industries as a source of secondary metabolites. Using MAPs and natural substances is one of the most important factors in ensuring human health. *Dracocephalum kotschyi* Boiss (locally called Badrandjboie‐Dennaie or Zarrin‐Giah) is a perennial aromatic plant belonging to the Lamiaceae family, native to Central Asia and Iran, where it usually grows in the highlands of Golestan, Mazandaran, Hamedan, Kermanshah, Fars, Tehran, and Semnan provinces (Fattahi, Nazeri, Torras‐Claveria, et al., 2013; Fattahi, Nazeri, Torras‐Claveria, et al., 2013). The aerial parts of *D. kotschyi* contain fragrant essential oil (EO), mainly composed of limonene, α‐terpineol, citral, α‐pinene, geranial, etc. This plant contains valuable compounds including methoxylated flavones (such as apigenin, luteolin, isokaempferid, crisimaritin, penduletin, and xanthomicrol) and phenolic compounds (such as rosmarinic acid, caffeic acid, chlorogenic acid, phenylpropanoids, and flavonoids (Heydari et al., 2019; Jahanian et al., 2005). *D. kotschyi* has antioxidant, anti‐inflammatory, antinociceptive, antibacterial, and antispasmodic activities and it can play an influential role in treating MS, cancer, dizziness, improving memory, glycemic, gastric ulcer, strengthening the immune system, and alleviating fever and rheumatism (Dastmalchi et al., 2007; Jahanian et al., 2005). Also, the dried *D. kotschyi* plants are used as herbal tea and flavor in foods.

Post‐harvest processing of MAPs is very essential due to the high sensitivity to fungal damage (caused by high moisture content). Therefore, moisture content should be reduced by 10%–12% during drying (Hazrati et al., 2021). Drying is one of the most widely used processes to protect foods against spoilage which reduces microbial and enzymatic activities and increases the product's shelf life. Drying facilitates the packaging, transportation, and storage of products by reducing their weight and volume (Li et al., 2011). There are two main drying methods: natural (sun and shade) and artificial (such as infrared, vacuum, and freeze drying; Mokhtarikhah et al., 2020). Despite many advantages such as low cost and simple technology, natural methods have several disadvantages. For example, it is prone to contamination and microbial spoilage during the process, it is time consuming, and it is climate dependent (Ebadi et al., 2015). Artificial drying methods are suitable alternative methods for MAPs drying but those should be considered carefully in terms of impact on active ingredients, cost, and energy consumption (Mokhtarikhah et al., 2020). In industrial processes, hot‐air drying is usually used for MAPs but it has some disadvantages, such as high energy consumption, long processing, and low quality of dried products (Ebadi et al., 2015). The suitable drying method should be selected based on the plant species, phytochemicals, phytochemicals storage place, and cost (Martin et al., 2017). The use of inappropriate methods can lead to undesirable changes in appearance, texture, color, and phytochemicals (Samani et al., 2017). Recently, innovative techniques such as infrared and refractance window drying have been reported that increase drying rates and maintain product quality.

Infrared (IR) drying is a combination of radioactive and conductive heating techniques used for heating, drying, and sterilization (Nozad et al., 2016). Once electromagnetic radiation (like infrared) hits the plant or food material, some of the radiation is absorbed and others are either reflected or transmitted. Absorbed radiation by material creates vibration in water molecules, so the material heats up and finally causes moisture evaporation and drying (Delfiya et al., 2021). Infrared drying (IRD) caused uniform heating, high drying rate, high quality of dried products, more energy efficiency, microbial decontamination, and sterilization. Due to the many advantages of IR drying, it is used for drying medicinal plants and food processing (Sakare et al., 2020). The positive role of IRD on MAPs has been reported by many researchers. Boateng et al. (2021) stated that IRD made ginkgoes more practical for the food industry by reducing their toxic compounds and ginkgolic acid. Moreover, the IR desiccant increased the content of pyridoxine, total phenol content, total flavonoid content and antioxidant activity in dried ginkgo compared to fresh herbs. IRD positively increased the amount of perilla aldehyde, D‐germacrene, and trans‐caryophyllene of *Dracocephalum kotschyi* essential oil and the highest IR wavelength level (0.5 w/cm) leading to the maximum production of these components (Samadi et al., 2018). In addition to shortening the drying time, IRD increased the EO content and composition of *Lippia citriodora* samples (Ebadi et al., 2016). It is reported that IRD reduced the microbial load of peppermint and cumin with no change in color and phytochemical compounds (Eliasson et al., 2014; Erdogdu & Ekiz, 2011).

Refractance window drying (RWD) is an innovative and energy‐efficient method (Baeghbali et al., 2020) that is appropriate for heat‐sensitive products. RWD‐dried products had high retention of sensory properties such as aroma, flavor, and color, as well as vitamins and antioxidants than other conventional methods (Baeghbali et al., 2020). Also, as the RWD has significant microbial inactivation capability, the dried product's safety will be increased (Nindo & Tang, 2007). This technology is relatively simple, inexpensive, and easy to use. Owing to the many benefits of this drying technology, it has found many applications in the pharmaceutical, food, and cosmetic industries (Ortiz‐Jerez et al., 2015). The positive effect of RWD has been shown in several studies. Drying bananas with RWD at 90°C caused the preservation of TPC, TFC, AOA, and ascorbic acid retention with minimum color change compared to the other conditions studied (Rajoriya et al., 2021). Drying apple slices with RWD and IRD+ RWD methods preserved taste, color, phenol, flavonoids, and antioxidant compounds, as well as increased energy efficiency and shortened drying time (Rajoriya et al., 2020). In other research, RWD increased the picrocrocin, safranal, crocin, and anthocyanin of saffron (Aghaei et al., 2018).

Due to the importance of the drying process in the MAPs industry and the usage of dried *D. kotschyi*, the present study was conducted for the first time to compare various drying methods (RWD, IRD, and RWD+ IRD in comparison to conventional drying methods) to select the appropriate method with an emphasis on protecting the phytochemical characteristics accompanied by reducing microbial contamination.

## 2 MATERIALS AND METHODS

### 2.1 Samples preparation

The *D. kotschyi* fresh plants were collected from the Bivarzin village (36°40′56″ N 49°34′43″ E and 1710 m altitude) in Amarlu district, Rudbar County, Guilan province, Iran.

### 2.2 Drying methods

#### 2.2.1 Natural drying

In shade drying (SHD), fresh plants were dried at room temperature (25°C) with natural airflow, and for sun drying (SD), the plants were dried under the sunlight at 36°C. Samples were weighed by digital balance (LutronGM‐300p) until they reached the final weight (10% of base moisture).

#### 2.2.2 Cabinet drying (CD)

In this method, a cabinet dryer (Artaco, Arvin Tabriz, Iran) equipped with a ventilation and air circulation system was used at two temperatures 50 and 60°C.

#### 2.2.3 Infrared‐assisted refractance window drying (RWD+ IRD) and refractance window drying (RWD)

**FIGURE 1:**
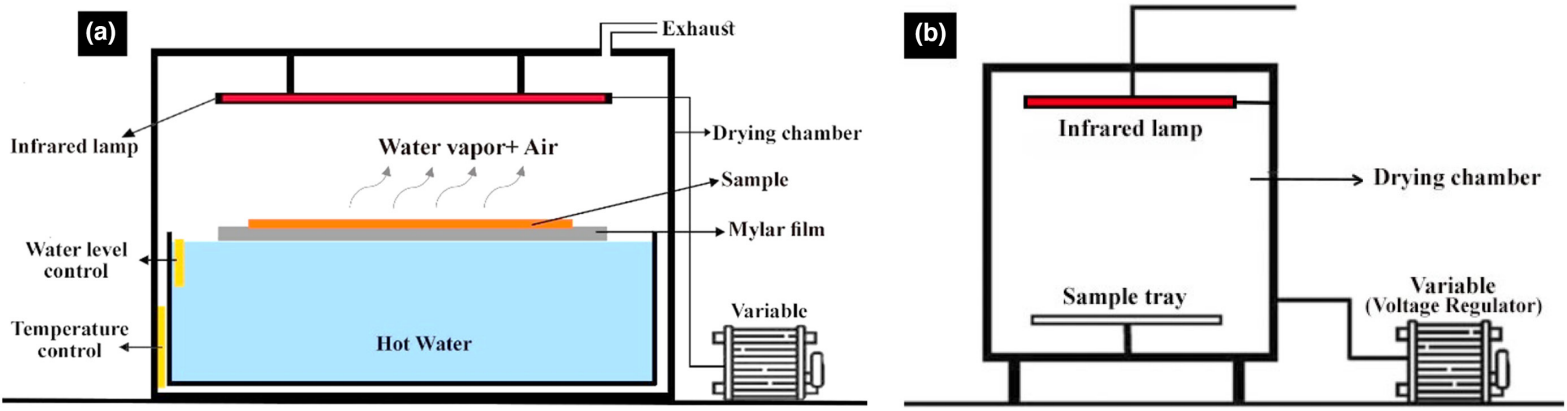
Experimental dryers. (a) Schematic diagram of IR‐assisted refractance window dryer (RWD + IRD) and refractance window dryer (RWD); (b) Schematic diagram of infrared dryer (IRD).

To dry plants by these methods, a laboratory‐scale infrared‐assisted refractance window dryer was used (Department of Food Process Engineering, Gorgan University of Agricultural Sciences and Natural Resources, Gorgan, Iran—Schematic IRD, Figure 1a). In this dryer, a Mylar film (DuPont) was placed on a hot water source at 90°C, and plant samples were spread on the surface of the Mylar film. The infrared lamp (NIR, Noor Lamp Company) was another main component of the dryer. In the RWD method, the IR lamp of the dryer was turned off, while in the combination method (RWD+ IRD), the IR lamp was turned on. For regulation of IR's power, a variable device (LS‐1P‐3K‐VA, Gold star) was used and RWD+ IRD 200 and 300 W were considered as combined treatments.

#### 2.2.4 Infrared drying (IRD)

In this experiment, a laboratory‐scale infrared dryer with a single‐sided radiation system (Department of Food Process Engineering, Gorgan, Iran—Schematic IRD, and Figure 1b) was used. An IR lamp (NIR, Noor Lamp Company) was placed above and at 15 cm from the sample tray. About 30 min before the drying process, the IR lamp was turned on with the required power so that the temperature conditions inside the chamber and the lamp temperature reach a stable state. The IR lamp's power was adjusted by a variable (LS‐1P‐3K‐VA, Gold star) and samples were dried at two powers (200 and 300 W).

### 2.3 Drying characteristics

#### 2.3.1 Moisture content

The fresh samples (50 g) were dried in a conventional oven (INB 400, Memmert) at 105°C for 24 h. Moisture content (based on fresh weight) was obtained using Equation (1):

(1) $$M_{w}=\frac{\left(W_{w}-W_{d}\right)}{W_{w}}\times 100,$$

*M*_w_: moisture weight based on fresh weight (%), *W*_w_: fresh weight (g), and *W*_d_: dry weight (g).

#### 2.3.2 Drying time

Drying time in each method was recorded based on the time (min) to reach the final weight (10% of base moisture).

#### 2.3.3 Drying rate

The drying rate was obtained by measuring the amount of weight loss during drying and reported as grams per hour.

### 2.4 Total phenol content

TPC was measured by the Folin–Ciocalteu method. First, 70 μL of the methanolic extract was mixed with 130 μL distilled water and 1 mL of 10% Folin, and kept at room temperature for 5–8 min, and then, 800 μL of sodium carbonate (7.5%) was added. The mixture was placed in a water bath at 40°C for 1.5 h in darkness and immediately read by spectrophotometer (T80+ UV/VIS Spectrophotometer, PG Instrument Ltd) at 760 nm. TPC was expressed in mg gallic acid equivalent per gram dry matter (mg GAE/g DM; Singleton et al., 1999).

### 2.5 Total flavonoid content

TFC was estimated by the aluminum chloride method (Du et al., 2009). So, 100 μL of the plant extract, 1750 μL of distilled water, 75 μL of sodium nitrite (0.5 M), and 75 μL of aluminum chloride (0.3 M) were mixed and after 5 min, 500 μL of NaOH (1 M) was added and kept in darkness for 15 min. The samples were read at 506 nm and TFC was expressed in mg quercetin equivalent per gram dry matter (mg QE/g DM).

### 2.6 Free radical scavenging activity

Free radicals scavenging activity (AOA) was calculated using the free radical DPPH method. Fifty microliter of the plant extract and 950 μL of DPPH 0.1 mM were placed in darkness for 15 min. Then, the sample was read at 517 nm (Chiou et al., 2007). The antioxidant capacity was calculated as the percentage of DPPH inhibition using Equation (2):

(2) $$\text{Free radical scavenging percentage}=\left(\left(A_{\text{control}}–A_{\text{sample}}\right)/A_{\text{control}}\right)\times 100.$$

### 2.7 Glandular trichome analysis by scanning electron microscopy (SEM)

The influence of drying methods on dried plants' microstructure was assessed by scanning electron microscopy. The samples were mounted on double‐sided carbon tape and coated with a thin layer of gold and scanned with an SEM (XL 30, Philips) at 25 kV.

### 2.8 EO content

To prepare EO, 20 g of the powdered plant was hydrodistillated by a Clevenger apparatus for 3 h. EO percentage was calculated by Equation (3):

(3) $$\mathrm{EO}\;\text{percentage}=\left(\mathrm{EO}\;\text{weight}/\mathrm{dry}\;\text{matter weight}\right)\times 100.$$

### 2.9 Color

#### 2.9.1 L*, a*, and b* color parameters

To measure the color parameters, 6 g of dried and powdered plants were poured evenly on a Petri dish and photographed by the camera (HS 20 EXR, Fujifilm) and analyzed by Color Average software to obtain the color parameters L* (lightness or brightness, 100: white and 0: black), a* (red/green value, positive is red and negative is green), and b* (blue/yellow value, positive is yellow and negative is blue).

#### 2.9.2 Chroma index

This parameter indicated the degree of saturation or color intensity and was calculated by the following equation (4):

(4) $$\text{Chroma}={\left(a^{2}+b^{2}\right)}^{1/2}$$

*a*: a* and *b*: b*.

#### 2.9.3 Hue angel index

It indicated the dominant color which was obtained by Equation (5):

(5) $$\mathrm{Hue}\;\text{angle}=\tan^{-1}\left(b/a\right)$$

*a*: a* and *b*: b*.

#### 2.9.4 Browning index (BI)

It showed the color changes of a plant to brown (Equations 6 and 7):

(6) $$\mathrm{BI}=\frac{\left(100-\left(x-0.31\right)\right)}{0.071}$$

(7) $$x=\frac{a+1.75L}{5.645\;L+a-3.012\;b}$$

*L*: L*, *a*: a*, and *b*: b*.

#### 2.9.5 Total color difference (Δ*E* index)

The Δ*E* index indicated the color changes during drying. The higher value indicated a greater difference with the control. Δ*E* index was obtained by Equation (8):

(8) $$\mathrm{\Delta E}=\sqrt{{\left(\mathrm{Lo}-L\right)}^{2}++{\left(\mathrm{ao}-a\right)}^{2}+{\left(\mathrm{bo}-b\right)}^{2}}$$

*L*_0_, *a*_0_, and *b*_0_: color indices before drying, *L*: L*, *a*: a*, and *b*: b*.

### 2.10 Microbial load

The microbial contamination was determined by dilution method and culture in specific media. First, 10 g of the plants were sterilized with 90 mL of buffer peptone solution in sterilized conditions. Then, a series diluted up to 10^−3^ was prepared. To determine aerobic mesophiles, plate count agar medium (PCA) was used. Mold and yeast were determined by yeast glucose chloramphenicol agar (YGCA) medium, and violet‐red bile lactose agar (VRBL) medium was used to determine coliforms. The samples were cultured directly in Petri dishes and kept at the appropriate incubation temperature (PCA at 30°C for 48 h, YGCA at 30°C for 72 h, and VRBL at 37°C for 24 to 48 h). After incubation, colonies were counted and reported based on log CFU in grams (dry weight) (Kneifel et al., 2002).

### 2.11 Statistical analysis

This experiment was conducted as a completely randomized design with nine drying treatments in three replications. Means were compared using the LSD test at *p* < 0.05 with SAS statistical software (SAS Institute, Cary, Ver. 9) and the graphs were drawn by MS Excel software. Hierarchical cluster analysis was performed using Euclidean distances with SPSS 22.0 Ver. statistical software.

## 3 RESULTS AND DISCUSSION

### 3.1 Drying time and rate

#### 3.1.1 Drying time

**FIGURE 2:**
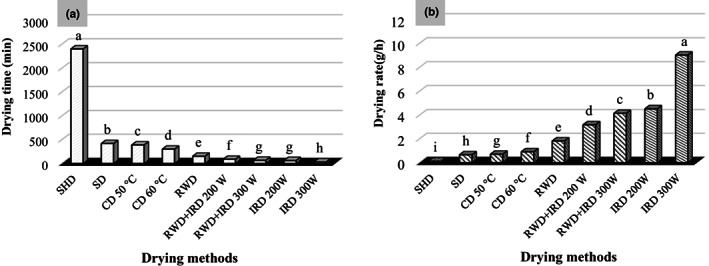
Drying time (a) and drying rate (b) of *Dracocephalum kotschyi* by different drying methods. CD, cabinet dryer; IRD, infrared dryer; RWD + IRD, combination method of refractance window and infrared dryers; RWD, refractance window dryer; SD, sun drying; SHD, shade drying.

Drying methods had a significant effect (*p* < 0.01) on drying time and rate (Table 1). IRD 300 W was the fastest drying method among the treatments and showed a significant difference from the control and others. In IRD 300 W, plants were dried for 30 min and followed by IRD 200 W, RWD+ IRD 300 and 200 W, and RWD significantly reduced moisture and dried plants for 60, 65, 85, and 150 min, respectively. A longer drying time (2400 min) was observed in the control sample (Figure 2a).

**TABLE 1:** ANOVA of drying methods on drying time, drying rate, and phytochemical compounds of *Dracocephalum kotschyi.*

Source of variation	df	Drying time	Mean of square				
			Drying rate	TPC	TFC	AOA	EO
Drying methods	8	1697606.25**	24.16**	0.26**	0.08**	4.29**	0.06**
Error	18	33.33	0.000	0.04	0.008	0.91	0.0005
CV	—	1.33	0.13	9.31	4.35	1.24	4.34

Abbreviations: AOA, antioxidant activity; CV, coefficient of variation; df, degree of freedom; EO, essential oil content; TFC, Total flavonoid content; TPC, Total phenol content.

** Significant at *p* < .01.

#### 3.1.2 Drying rate

The highest drying rate (9.01 g/h) observed in IRD 300 W had significant difference with control (0.11 g/h). IRD 200 W showed the highest drying rate. RWD and RWD+ IRD also effectively increased the drying rate compared to the control and other conventional methods (Figure 2b).

The preservation of the qualitative characteristics of MAPs in the post‐harvest stage is one of the main concerns of MAPs production and processing industries (Ebadi et al., 2015). Drying is used to reduce the moisture content of foodstuffs to reduce the microbial spoilage speed and chemical changes, increasing product shelf‐life and reducing weight and required space (Sui et al., 2014). The time required to dry plant samples depends on the material's volume and moisture content as well as drying temperature. As shown, the most prolonged time and the lowest drying rate were observed in the control. Similarly, Mokhtarikhah et al. (2020) reported that the longest drying time (28 h) was recorded in shade‐dried mint. Drying the *Clinacanthus nutans* leaves at higher IR power increased the drying rate and reduced drying time (Abdullah et al., 2022). In *Artemisia absinthium* L., drying duration decreased by increasing IR power (Beigi, 2018). Also, in *Stevia Rebaudiana* (Bakhshipour et al., 2020) and *Mentha spicata* L. (Nozad et al., 2016), drying time decreased by increasing the drying temperature and IR radiation. These results are in accordance with our findings that the shortest duration time and highest drying rate of the *D. kotschyi* plant were observed in IRD, especially in high power. High IR power increases vapor pressure in the samples and causes faster moisture removal from inside to the product's surface. Also, higher temperatures improve heat transfer between thermal source and drying materials, accelerate moisture evaporation from the material's surface, and reduce drying time (Delfiya et al., 2021). Reducing drying time is very effective in reducing energy costs (HamrouniSellami et al., 2011), so the use of IRD can be effective in reducing the cost of *D. kotschyi* production. Aghaei et al. (2018) reported that RWD at 80°C was the best treatment for drying saffron stigmas. In *physalis*, the drying time noticeably decreased by increasing the temperature from 60 to 95°C (Puente‐Díaz et al., 2020). These results were consistent with the positive effect of the RWD on the drying time in the current study. Drying time reduction may be related to the specific mechanism of RWD and heat transfer by conduction and radiation. The RWD causes faster water distribution by facilitating the porous structure formation (Rajoriya et al., 2020). Overall, using IR and RWD as synergy emerging technology for drying fruit, vegetable, and other food on an industrial scale could be considered for reducing the drying time and energy consumption (Puente‐Díaz et al., 2020).

### 3.2 Total phenol content (TPC)

**FIGURE 3:**
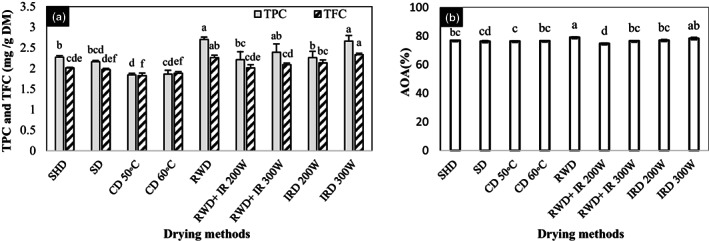
(a) Total phenol content (TPC) and total flavonoid content (TFC); and (b) antioxidant activity (AOA) of *Dracocephalum kotschyi* by different drying methods. CD, cabinet dryer; IRD, infrared dryer; RWD + IRD, combination method of refractance window and infrared dryers; RWD, refractance window dryer; SD, sun drying; SHD, shade drying.

According to Table 1, drying methods significantly affected the TPC and TFC of *D. kotschyi*. The maximum TPC (2.7 mg GAE/g DW) was observed in samples dried by RWD, but it had no significant difference from IRD 300 W (2.66 mg GAE/g DW) and RWD+ IRD 300 W (2.39 mg GAE/g DW). In these methods, the TPC was significantly increased compared to the control. The minimum TPC (1.84 and 1.86 mg GAE/g DW) was observed in CD 50 and 60°C (Figure 3a).

Phenolic compounds are a large group of secondary metabolites with antioxidant properties with wide distribution in plants. The antioxidant properties of phenolic compounds are attributed to the aromatic and hydroxyl groups in their structures (Duc Pham et al., 2019). Phenolic compounds are affected by various factors such as drying (Uddin et al., 2022). Cheng et al. (2019) reported that dried coffee beans had more TPC than fresh samples. Drying apple slices with RWD increased TPC by 10.6%–14% compared to hot‐air drying which may be attributed to rapid heating that releases phenolic compounds from the apple cell matrix (Rajoriya et al., 2020). Also, RWD effectively increased the TPC in Bananas (Rajoriya et al., 2021), mango (Shende & Datta, 2020), and carrots (Hernández‐Santos et al., 2016). The increase in TPC of *D. kotschyi* by the RWD method was consistent with these studies which can be due to the formation of phenolic compound precursors with non‐enzymatic conversion between phenolic molecules during drying (Vega‐Gálvez et al., 2009). The TPC of garlic slices dried by IRD significantly increased by increasing the temperature from 50 to 80°C at 1575 W (Zhou et al., 2016). The effect of high IR power on increasing the TPC in the current study was consistent with this report. An increase in the TPC of dried samples with increasing IR power is probably related to the ability of IR radiation to break covalent bonds and release polyphenols (Lee et al., 2003). In the current study, a high amount of TPC was observed in samples dried by RWD+ IRD 300 W. Similarly, the TPC of apples dried by RWD+ IRD 50 and 60°C was increased compared to the hot air dryer (Rajoriya et al., 2020). According to reports, the increase in phenolic compounds in drying conditions may be due to several reasons, and prolonging drying time and high temperature might destroy the cellular structure and further release some bound phenolic compounds (Zhou et al., 2016). Also, some phenolic compounds were produced due to the structural transformation of polyphenols during high drying temperatures (Lopez et al., 2010). On the other hand, the formation of phenolic compounds could be due to the availability of phenolic precursors through non‐enzymatic interconversion (Que et al., 2008; Zhou et al., 2016).

### 3.3 Total flavonoid content (TFC)

The maximum TFC (2.33 mg catechin/g dry weight) was obtained from the plants dried by IRD 300 W that had no significant difference with RWD (2.26 mg catechin/g dry weight). Also, RWD+ IRD increased the TFC of dried plants (Figure 3a). The minimum TFC was observed in CD 50 and 60°C (1.82 and 1.88 mg catechin/g dry weight, respectively).

Flavonoids are the largest group of polyphenols that have antioxidant activity and inhibition of free radicals. Flavonoids have hepatoprotective, anti‐inflammatory, anticancer effects, etc. Also, these compounds have the ability to prevent cardiovascular disease (Wu et al., 2023). The positive impact of IR in this study was consistent with some research. Boateng et al. (2021) stated that IRD increased TFC in dried ginkgoes compared to fresh herbs. In potatoes, the TFC in the medium‐frequency ultrasound device (40 kHz) was the highest at a lower IR temperature (60°C) as compared to higher temperatures (80°C). The amounts of rutin, quercetin 3‐β‐D‐glucoside, and other flavonols were significantly higher in the ultrasound‐treated samples at a low frequency (20 kHz) at an average temperature of IR (Rashid et al., 2019). In sage, the maximum TFC was obtained from plants dried with IRD 65°C (HamrouniSellami et al., 2013). The TFC of *B. subtilis*‐fermented polished adlay dried by IRD was 1.60 times higher than that of freeze‐vacuum drying samples (Wen et al., 2020). Also, drying *D. kotschyi* by RWD and RWD+ IRD increased the TFC. Talukdar and Uppaluri (2021) reported that the turmeric dried by RWD at 95°C had the maximum TFC. Since the high temperature of the RWD reduced the drying time, the samples were exposed to heat for a shorter time, and consequently, retained heat‐sensitive constituents. Due to the fast drying time, the cellular integrity is not compromised, and the TFC in the cell structure is not oxidized but preserved (Hernández‐Santos et al., 2016; Talukdar & Uppaluri, 2021). The TFC in dried apples by RWD and RWD+ IRD was higher than in the hot‐air dryer. The combination methods can prevent the degradation of flavonoids due to a significant reduction in drying time (Rajoriya et al., 2020).

### 3.4 Antioxidant activity (AOA)

Based on Table 1, drying methods significantly affected the AOA of *D. kotschyi* (*p* < 0.01). The maximum AOA was estimated at 79% in the plants dried by RWD. Also, using IRD 300 W increased the AOA (78.33%). Both methods outperformed the control. The minimum AOA was observed in RWD+ IRD 200 W which had no significant difference with SD method (76.27%) (Figure 3b).

Antioxidants inactivate the free radicals and protect the cells from destructive effects (Nora et al., 2014). Many researchers stated that high AOA in fruits and vegetables after drying might be due to the more antioxidant activity of oxidized polyphenols than non‐oxidized polyphenols (Nora et al., 2014; Önal et al., 2019). In addition, an increase in AOA after drying may be due to Millard reaction products (MRPs) which are caused by heat treatment or long‐term storage and generally show strong antioxidant properties (Kamiloglu & Capanoglu, 2014; Önal et al., 2019). According to our findings, the maximum AOA was observed in RWD‐dried plants. A similar result was reported by Nansereko et al. (2021) that the RWD effectively increased Jackfruit's antioxidant activity. Rajoriya et al. (2021) stated that the highest level of AOA preservation was observed in bananas dried by RWD at 90°C. It may be due to the rapid moisture decreasing at higher temperatures, which minimizes the degradation of antioxidant compounds and increases the release of restricted phenolics from the cellular matrix (Hernández‐Santos et al., 2016; Zhou et al., 2016). RWD and RWD+ IRD had significant antioxidant activities compared to hot‐air dryers (Rajoriya et al., 2020). In saffron stigma, the maximum antioxidant compounds such as crocin, picrocrocin, and anthocyanin were observed in RWD at 70 and 80°C (Aghaei et al., 2018). IRD is an effective technique to increase the products' oxidative capacity. The *B. subtilis*‐fermented polished adlay dried by hot air and infrared had AOA significantly higher than under vacuum, freeze‐vacuum, and microwave‐vacuum drying methods (Wen et al., 2020). Similarly, our findings showed that IRD 300 W increased the plant's AOA. Zhou et al. (2016) stated that AOA in garlic slices increased during IRD, which is probably due to the IR's ability to break covalent bonds and release antioxidants. The highest DPPH antioxidant activity was obtained from sweet potatoes dried by IRD (>80°C) and the increase after drying can be related to the increase in total phenols, flavonoids, and other phytochemical compounds released from the cell (Rashid et al., 2019).

### 3.5 Glandular trichomes

**FIGURE 4:**
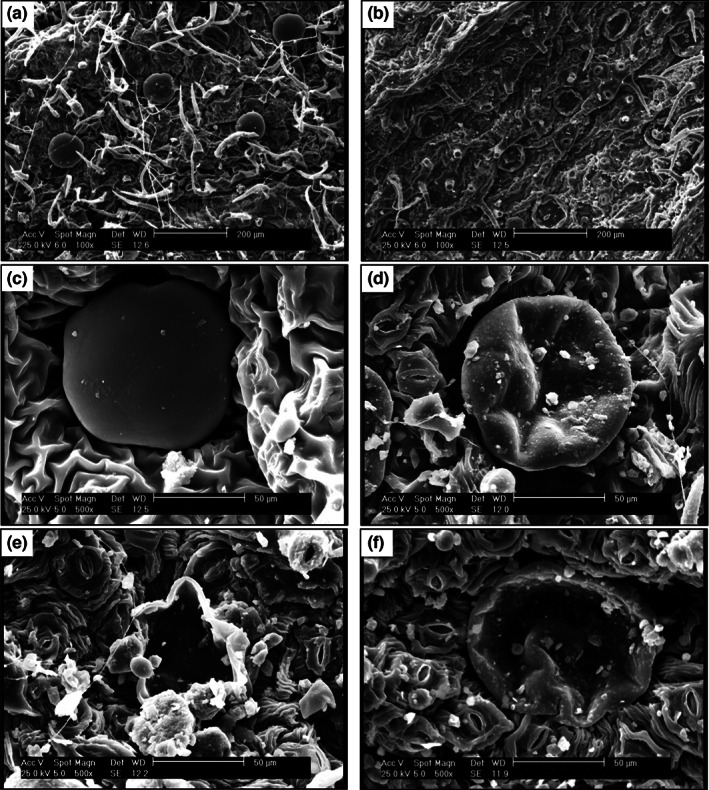
SEM micrographs from glandular trichomes of *Dracocephalum kotschyi*. (a) Distribution of glandular trichomes on leaf lower surface (abaxial); (b) glandular trichomes in IRD 300 W; (c) intact glandular trichomes in the fresh sample (before drying); (d) damaged glandular trichomes by SHD; (e) ruptured glandular trichomes by CD 50°C; and (f) damaged glandular trichomes by RWD. Scale: a, b = 200 μm, c–f = 50 μm. CD, cabinet dryer; IRD, infrared dryer; RWD + IRD, combination method of refractance window and infrared dryers; RWD, refractance window dryer; SD, sun drying; SHD, shade drying.

Trichomes are specialized structures with many morphologies and functions that initiate from young epidermal cells and cover aerial tissues (Feng et al., 2021). Glandular trichomes as the storage and secretion site of EOs are widely distributed in the Lamiaceae family. Thereby, the evaluation of glandular trichome changes during post‐harvest processes is important for their impact on EO content (Cappellari et al., 2019; Mokhtarikhah et al., 2020; Nazari et al., 2018). SEM images of *D. kotschyi* (Figure 4) showed that glandular trichomes are vulnerable to drying conditions. The deformation of trichomes in some methods was obvious. Rupturing of the secretory structure in samples dried by CD 50°C can be due to long‐term exposure to the dryer temperature. The minimum changes and damages in the secretory trichomes were observed in SHD. Our findings about the inappropriate effects of drying methods on glandular trichomes were in accordance with some studies. In spearmint, some methods such as vacuum, microwave, and oven drying with temperatures less than 50°C caused trichomes to break or wrinkle (Mokhtarikhah et al., 2020). In lemon verbena, glandular trichomes were damaged by increasing temperature and time prolongation in the oven and vacuum dryer, respectively (Ebadi et al., 2015). An evident collapse and shrinkage in the *chrysanthemum* structure dried by IR‐HAD was observed which could be due to the different vapor pressure between the outside and inside of samples during drying (Xu et al., 2022). An et al. (2016) reported that the microstructures of ginger IR dried were well preserved and less damaged due to the less heating and shorter duration of IR radiation, which is in accordance with our observations on glandular trichomes of the IR‐dried plants compared to CD.

### 3.6 EO content

**FIGURE 5:**
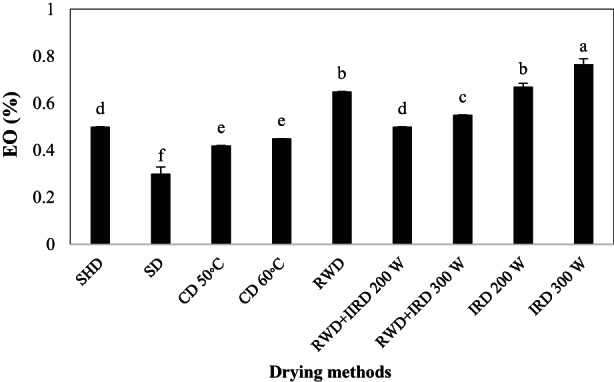
Essential oil content (EO) of *Dracocephalum kotschyi* by different drying methods. CD, cabinet dryer; IRD, infrared dryer; RWD + IRD, combination method of refractance window and infrared dryers; RWD, refractance window dryer; SD, sun drying; SHD, shade drying.

Drying methods had a significant effect on the EO content of *D. kotschyi* (Table 1). As shown in Figure 5, the maximum EO content (0.76%) was obtained from IRD 300 W followed by IRD 200 W and RWD (0.67% and 0.65%, respectively). The minimum EO content was observed in the SD and CD 50°C method.

EOs are a group of secondary metabolites that are influenced by post‐harvest processing. EOs' quantity and quality depend on the drying duration, drying method, and plant species (Yazdani et al., 2006). HamrouniSellami et al., 2011 stated that the EO content and 1, 8‐cineole of *Laurus nobilis* were increased by IRD at 45°C. Also, IRD had a better EO yield compared to the oven dryer (at the same temperature) which could be related to the drying time. In the oven dryer, plant material dried for several days, so EO evaporation in the atmosphere dryer was higher than IR drying which dried in a few hours (HamrouniSellami et al. (2011)). This result approved our findings that long‐term exposure to temperature in CD 50°C caused trichome rupturing and EO reduction compared to IRD. In the current study, IRD at higher power was more effective on the EO content. Similarly, Ebadi et al. (2016) stated that increasing the IR intensity at all air flow rates caused an increase in the EO percentage of *Lippia citriodora*. MAP species, EO storage place, the type of EO components, and other factors provoke different responses to different intensities of IR. RWD increased the EO content of *D. kotschyi* which was consistent with another study on saffron. Aghaei et al. (2018) reported that the safranal content (as saffron aroma factor) was increased in the RWD method at 70 and 80°C. The quality of food products obtained by RWD is better than the other dryers. Quick and gentle drying in RWD minimizes heat‐induced degradation and oxidation and maximizes product fragrance and flavor (Bernaert et al., 2019). An increase in the EO content in some drying methods (e.g., IRD and RWD in the current study) may be due to the gradual stress in plants (Mokhtarikhah et al., 2020). Respiration of plants continues until thoroughly dry, so they try to preserve their intracellular balance during drying through the activation of internal mechanisms. Therefore, the plant may prevent water loss by producing active ingredients such as EO (Castelló et al., 2006; Lewicki et al., 2001; Mokhtarikhah et al., 2020). Generally, the increase or decrease in EO content during drying process may be related to differences in plant species, glandular trichomes structure, and EO chemical components (HamrouniSellami et al. (2011)).

### 3.7 Color

**FIGURE 6:**
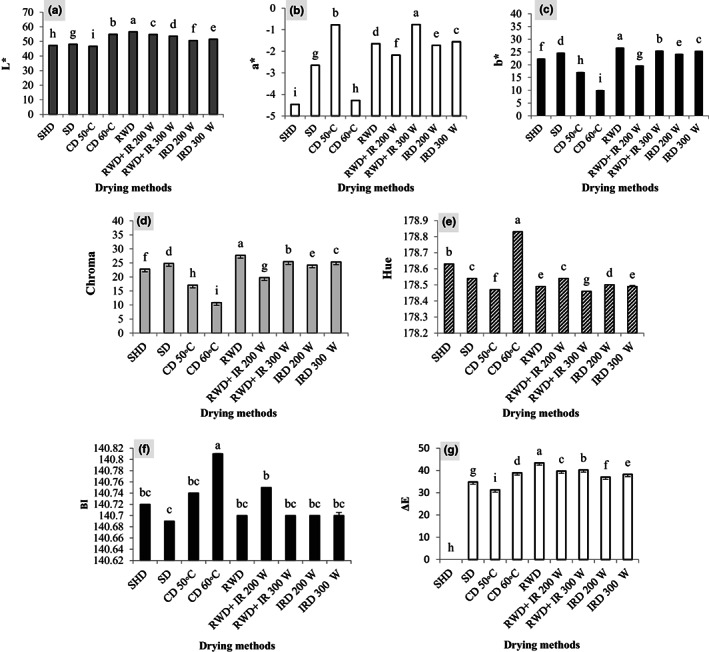
Color changes of *Dracocephalum kotschyi* by different drying methods. (a) L* (brightness); (b) a* (red/green value); (c) b* (blue/yellow value); (d) chroma (color intensity); (e) hue (dominant color); (f) BI (browning index); (g) Δ*E* (total color changes). CD, cabinet dryer; IRD, infrared dryer; RWD + IRD, combination method of refractance window and infrared dryers; RWD, refractance window dryer; SD, sun drying; SHD, shade drying.

As shown in Table 2, the drying methods significantly affected the color parameters (*p* < 0.01). The highest and lowest L* index (56.52 and 46.65) was obtained from RWD and CD 50°C, respectively. The IRD and RWD+ IRD methods were also at a high level of the L* index and showed a significant difference compared to the control (Figure 6a). The control (−4.46) and CD 60°C (−4.28) had the highest a* index while the lowest was observed in RWD+ IRD 300 W (0.76) and CD 50°C (−0.77). RWD+ IRD 200 W, RWD, and IRD independently had an acceptable level of a* index (Figure 6b). According to Figure 6c, the highest amount of the b* index was obtained from the RWD method (25.56), followed by RWD+ IRD 300 W (25.46), IRD 300 W (25.35), and IRD 200 W (24.19), which were significantly different from the control. The lowest amount of the b* index was observed in the plants dried by CD 60°C (10) and 50°C (17.07). The highest chroma index was observed in the RWD‐dried samples (27.7). Also, RWD+ IRD 300 W (25.47), IRD 300 W (25.39), and IRD 200 W (24.25) had a high chroma index. All of these methods had a higher chroma index than the control. The lowest value of chroma index was observed in the plants dried by CD at 60°C (10.87) (Figure 6d). Based on Figure 6e, the plants dried by CD 60°C had the maximum hue index. Other treatments such as RWD, IRD 300, and 200 W had medium and RWD+ IRD 300 W had the minimum hue index. The highest browning index (140.81) was observed in CD 60°C method. The lowest amount of browning was observed in the SD samples (140.69), which had no significant difference from the others. Also, BI index had low value in RWD, IRD, and RWD+ IRD methods (Figure 6f). As shown in Figure 6g, RWD‐dried plants (43.4) had the highest amount of Δ*E* and significantly differed from the control. RWD+ IRD 200 and 300 W and IRD showed a significant difference in color with the control.

**TABLE 2:** ANOVA of drying methods on the color indices and microbial load of *Dracocephalum kotschyi*.

Source of variation	df	L*	Mean of square							Mold & Yeast	Coli form
			a*	b*	Chroma	Hue	BI	Δ*E*	Aerobic mesophiles		
Drying methods	8	40.004**	5.5**	86.24**	79.53**	0.04**	0.004**	517.54**	0.022**	0.95**	0.22**
Error	18	0.00002	0.00001	0.00001	0.001	0.000	0.001	0.000	0	0.0001	0.00001
CV	—	0.009	0.14	0.015	0.15	0.002	0.02	0.01	0	2.64	0.18

Abbreviations: a*, red/green value; b*, blue/yellow value; chroma, color intensity; hue, dominant color; BI, browning index; CV, coefficient of variation; df, degree of freedom; L*, Brightness; Δ*E*, total color changes.

** Significant at *p* < .01.

Color is one of the most important appearance attributes and a significant quality index to assess thermal damage that directly affects consumer acceptability and marketability of dried products (Aral & Beşe, 2016; Maskan, 2001). Thus, the color indices were used to evaluate the changes in the color characteristics of the dried products. Alteration of the L*, a*, and b* index occurs due to pigment decomposition during drying, which can increase the value of Δ*E* and BI and ultimately reduce the product quality (Maskan, 2001). Drying conditions affected color changes and quality. The methods with high drying rates could preserve color by inhibiting the destruction of the pigments. In blackberry, the best color quality was obtained by microwave at higher temperatures, which means the fast‐drying process leads to better color preservation (Kaveh et al., 2020). In‐shade drying led to rapid degradation of photosynthetic pigments due to the longer drying process, which was consistent with results on savory and thyme (Rahimmalek & Goli, 2013). According to the results on dried bananas, the highest values of L*, b*, chroma, and Δ*E* along with the lowest values of a*, hue, and browning index were observed in the RWD method. L*, a*, and b* indices increased with increasing RWD temperature (Rajoriya et al., 2021). The L*, a*, and b* indices of cherries, strawberries, and cranberries dried by RWD, and freeze‐drying have been maintained and improved which is related to less decomposition of pigments during drying owing to mild drying conditions compared to hot air (Nemzer et al., 2018). Topuz et al. (2009) reported that RWD preserved the pepper color better than hot‐air drying because of less oxidation and decomposition of pigments. Rajoriya et al. (2020) stated that the highest L* index in apple fruit was obtained from the combined method (RWD+ IRD 60°C) which was higher than all other methods. IRD independently and in combination with RWD effectively maintained and increased the color indices of dried plants. It may be related to the efficient heat transfer in RWD and the combined method (RWD+ IRD), which leads to faster drying and prevents color degradation (Rajoriya et al., 2020). Similar to our results, it was reported by Aidani et al. (2016) that Δ*E* and L* values in kiwifruits were increased by increasing IR power from 200 to 300 W. IR can preserve the color properties of MAPs by reducing the drying time and limiting browning enzymatic interactions (Ebadi et al., 2016). Research on jujube has shown that Δ*E* of the dried samples improved with increasing IR levels. To reduce phenolic degradation and color changes and increase the dried product quality, Jujube fruits should be dried at 88 W before processing (Doymaz et al., 2016). Generally, RWD, IRD, and RWD+ IRD methods improved the color quality of the *D. kotschyi* dried and they had high color purity, acceptable green color, and less browning.

### 3.8 Microbial load

**FIGURE 7:**
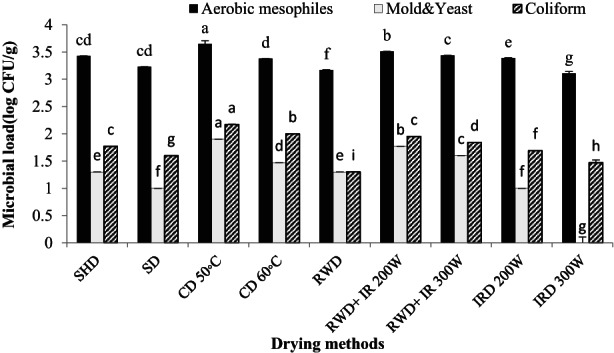
Microbial load of *Dracocephalum kotschyi* by different drying methods. CD, cabinet dryer; IRD, infrared dryer; RWD + IRD, combination method of refractance window and infrared dryers; RWD, refractance window dryer; SD, sun drying; SHD, shade drying.

**FIGURE 8:**
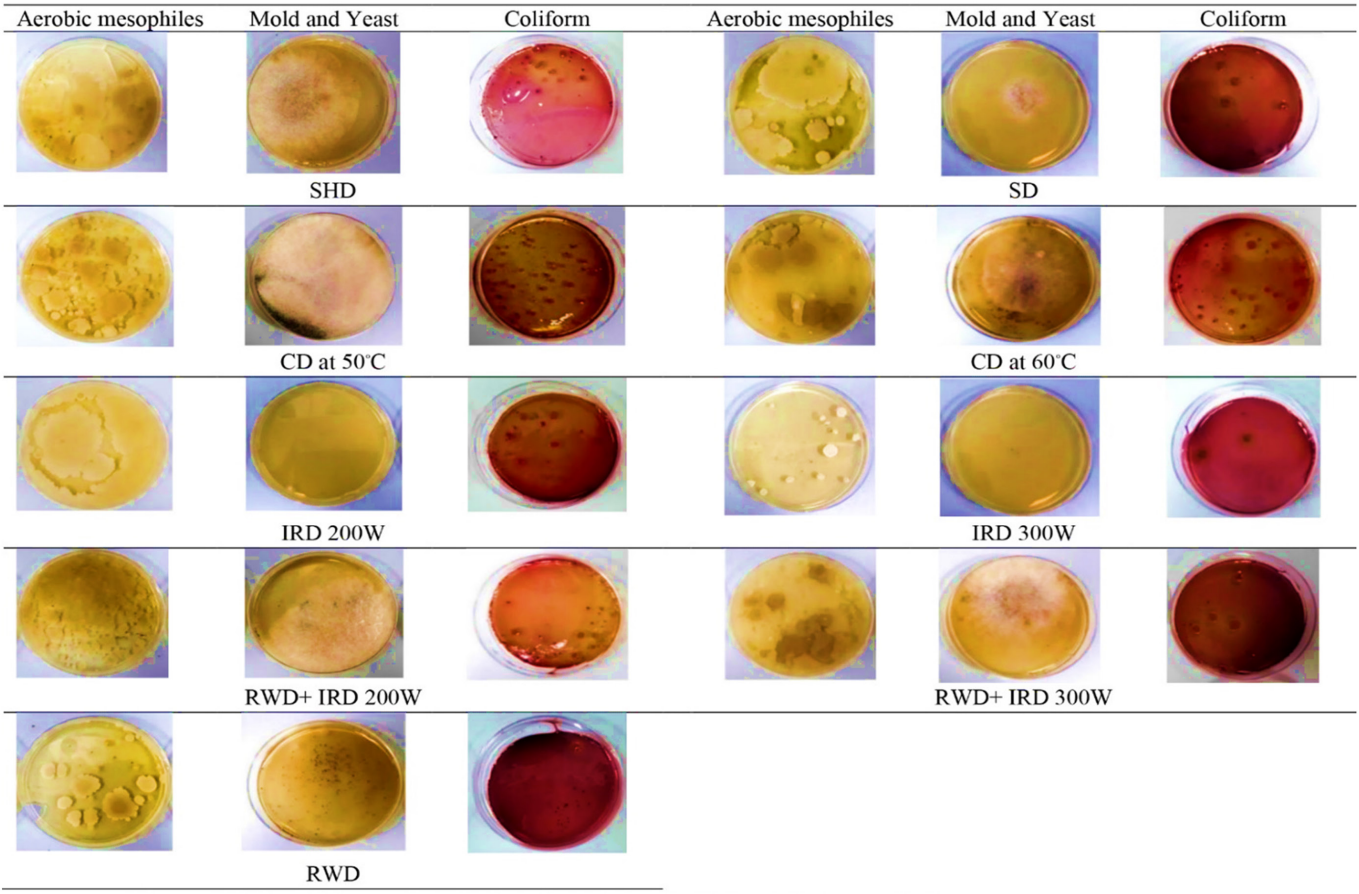
Microbial growth in *Dracocephalum kotschyi* dried by different drying methods. CD, cabinet dryer; IRD, infrared dryer; RWD + IRD, combination method of refractance window and infrared dryers; RWD, refractance window dryer; SD, sun drying; SHD, shade drying.

Based on Table 2, the effect of different drying methods on the microbial load of *D. kotschyi* was significant (*p* < .01). The maximum aerobic mesophiles, mold and yeast, and coliforms were observed at 3.65, 1.9, and 2.17 log CFU/g in the plants dried by CD 50°C, respectively. The minimum number of aerobic mesophiles was observed in IRD 300 W (3.11 log CFU/g) and RWD (3.17 log CFU/g) which showed less microbial load than the control. The combined method of RWD+ IRD was effective in reducing the plant's microbial load. No mold and yeast grew in the plants dried by IRD 300 W. Subsequently, the plants dried at IRD 200 W and SD had the minimum amount of mold and yeast contamination (1 log CFU/g). Slight contamination was observed in the plants dried by RWD and control (1.30 log CFU/g). According to Figure 7, the minimum coliforms (1.30 log CFU/g and 1.47 log CFU/g) were recorded in plants dried by RWD and IRD 300 W, respectively. Generally, RWD and IRD were more effective in reducing the microbial contamination of *D. kotschyi* than the control and other methods (Figures 7 and 8).

The quality, health, and efficiency of MAPs are essential factors that have drawn much attention. One of the standards defined by the World Health Organization (WHO) is related to the microbial load of MAPs (Mimica‐Dukic et al., 2004). During growth, harvest, and processing, MAPs are susceptible to microbial contamination due to the presence of contaminants such as soil, insects, and water (Eliasson et al., 2014). Pathogenic microorganisms such as *Salmonella*, *Escherichia coli*, *Bacillus cereus*, *Clostridium perfringens*, and toxigenic molds and yeasts have been detected in MAPs that could cause disease (Banerjee & Sarkar, 2003; Sagoo et al., 2009). Consequently, the decontamination of MAPs for pathogenic microorganisms' removal and prevention of food spoilage and food‐borne diseases is very important (Eliasson et al., 2014; Erdogdu & Ekiz, 2011). In addition, microbial contamination in plant products has limited their usage in the pharmaceutical, food, cosmetic, and health industries. Therefore, to use MAPs as food or herbal medicines, they should be adequately monitored to minimize the side effects of allergenic reactions and contaminants and to provide safe, efficient, and standard products to the consumer (Mimica‐Dukic et al., 2004).

Based on the results, RWD, IRD, and RWD+ IRD were more effective in reducing microbial contamination than other treatments. Among the electromagnetic radiations used for the combined drying method, IR is chosen due to its fast and uniform heating characteristics (Zeng et al., 2019). IR heating is a thermal technology and therefore it is presumed to inactivate microorganisms by heat. IR heating resulted in external and internal damage to the microorganisms' cells (damage to cell walls, shrinkage of cytoplasmic membranes, leakage of intracellular components, and thermal denaturation of proteins and nucleic acids) (Ramaswamy et al., 2012). However, as the IR spectrum lies between microwaves and UV light, an overlapping effect involving induction heating and damage to DNA has also been reported to be responsible for microbial inactivation (Krishnamurthy, 2006). Mirmostafaee et al. (2014) observed that the microwave method (compared to shade and oven at 45°C) had the most significant effect on reducing the fungal and aerobic mesophiles contamination. Microwave radiation led to the reduction in the microorganism's growth by destroying the cell membrane and structures. Also, the high drying rate of this method prevented the microorganism's growth (Mirmostafaee et al., 2014). IR can disinfect and reduce the microbial load of agricultural products, MAPs, and foods (Delfiya et al., 2021). IR heat reduced the microbial contamination caused by *Bacillus cereus* without changing the color and essential constituents of *Origanum vulgare* EO (including carvacrol and thymol) (Eliasson et al., 2014). According to Erdogdu and Ekiz (2011), IR disinfected and reduced aerobic mesophilic bacteria and eliminated mold and yeast, while no change was observed in the amount of EO and pigments of the cumin seeds. RWD technique, due to heat transfer by both radiation and conduction, in addition to shortening the drying time, is effective on the final quality of dried plants in terms of color, nutritional value, and microbial load (Nindo & Tang, 2007; Tontul & Topuz, 2017). RWD had a strong inactivation effect against aerobic mesophyll, coliforms, *Escherichia coli*, and *Listeria nomoa* of pumpkin purees that can be due to damage to the cell membrane and DNA of pathogenic microorganisms during the drying period (Nindo et al., 2003). In the RWD method, the low thickness and high transparency of the Mylar film allow IR rays to pass, so that more radiation is transferred to the product, hence it is effective in reducing the microbial load (Nindo & Tang, 2007). Here, the CD 50°C method did not successfully reduce the microbial load. Similarly, Mirmostafaee et al. (2014) reported that the most bacterial contamination was observed in the oven at 45°C. Since the oven had a closed environment with limited ventilation, the water vapor increased at the first hours of drying and by condensation of water, favorable conditions for the growth and reproduction of microorganisms were provided. On the other hand, the oven temperature (45°C) was not efficient to prevent bacteria growth.

**FIGURE 9:**
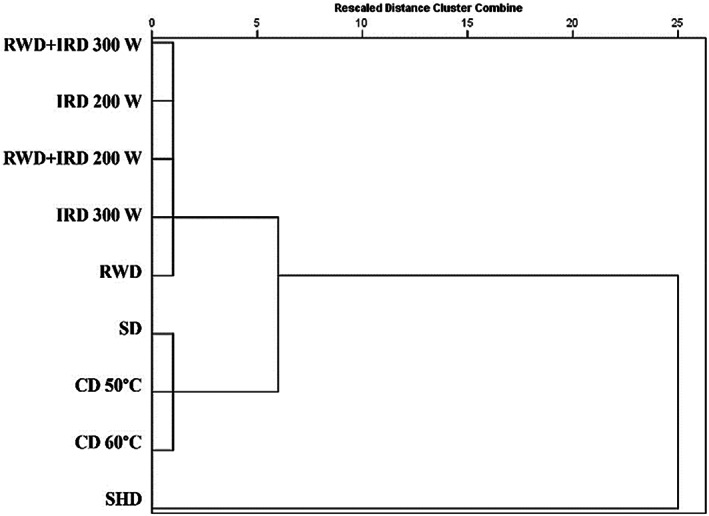
Dendrogram obtained by hierarchical cluster analysis of all traits in *Dracocephalum kotschyi* dried by different methods. CD, cabinet dryer; RWD + IRD, combination method of refractance window and infrared dryers; IRD, infrared dryer; RWD, refractance window dryer; SD, sun drying; SHD, shade drying.

Cluster analysis was applied to determine the relationship between the different drying methods based on all studied traits. A dendrogram of cluster analysis separated the drying methods into three major groups that were distinguished both in quality and quantity (Figure 9). Group 1, included the IRD (200, 300 W), RWD, and combination method (RWD+ IRD 200 and 300 W). Conventional drying methods such as CD 50, 60°C, and SD were placed in group 2. The SHD method was placed in a separate group (group 3). According to previous studies, the classification of different drying methods was affected by various factors. In *Lippia thymoides* and *Stachys lavandulifolia*, differences in EO components led to the grouping (Hazrati et al., 2021; Nascimento et al., 2021) while in *Thymus daenesis*, drying temperatures were the main factor of the grouping (Rahimmalek & Goli, 2013).

## 4 CONCLUSION

IRD, RWD, and IRD+ RWD significantly reduced the drying time compared to all treatments. In these methods, the *D. kotschyi* plants were dried 16 to 80 times faster than the control. Among the drying methods, IRD, RWD, and IRD+ RWD had a positive effect on maintaining qualitative characteristics. The maximum total phenol content (2.7 and 2.66 mg GAE/g dry weight), total flavonoid content (2.26 and 2.33 mg QE/g dry weight), antioxidant activity (79% and 78.33%), and EO content (0.65% and 0.76%) were reported in RWD and IRD methods, respectively. Evaluation of color indices revealed that plants dried by RWD, IRD, and RWD+ IRD were better than other treatments. In these methods, dried plants had high color purity and quality, acceptable green color, and less browning. The microbial load was also measured as an important indicator in determining the product's health for the consumers. Our results revealed that RWD and IRD were more effective in reducing microbial contamination than the control. IRD 300 W completely inactivated the mold and yeast growth. Also, the combined method of RWD+ IRD had a positive effect in reducing the plant's microbial contamination. Plants dried by CD at 50°C had the maximum microbial load. As shown in obtained results and dendrogram, there was a distinction between modern and conventional drying methods. Generally, to produce dried *D. kotschyi* with less time, more active substances content, high and acceptable color quality, and less microbial contamination is preferred to use modern drying methods such as RWD, IRD, and combination RWD+ IRD instead of conventional methods (SHD, SD, and CD).

## AUTHOR CONTRIBUTIONS

**Sahar Zamani:** Data curation (lead); investigation (equal); software (supporting); writing – original draft (supporting); writing – review and editing (equal). **Davood Bakhshi:** Funding acquisition (supporting); investigation (equal); project administration (supporting); supervision (equal); writing – review and editing (equal). **Amir Sahraroo:** Investigation (equal); supervision (equal); writing – review and editing (equal). **Mohammad‐Taghi Ebadi:** Data curation (equal); investigation (equal); software (supporting); supervision (equal); writing – review and editing (equal).

## CONFLICT OF INTEREST STATEMENT

The authors declare no conflict of interest.

## ETHICS STATEMENT

Our research did not contain any animal experiments or human subjects.

## Data Availability

The data that support the findings of this study are available from the corresponding author upon reasonable request.
